# DNA Transfer Between Items Within an Evidence Package

**DOI:** 10.3390/genes16080894

**Published:** 2025-07-28

**Authors:** Yong Sheng Lee, Christopher Kiu-Choong Syn

**Affiliations:** DNA Profiling Laboratory, Biology Division, Health Sciences Authority, 3 Biopolis Drive, Synapse, 03-14/15, Singapore 138623, Singapore; christopher_syn@hsa.gov.sg

**Keywords:** forensic DNA, DNA transfer, touch DNA, evidence packaging

## Abstract

**Background/Objectives:** Advancements in DNA profiling have made it possible to retrieve intact DNA profiles from increasingly minute biological samples. This increased sensitivity in DNA detection has highlighted crucial considerations to be made when handling and packing items from the crime scene to minimize potential contamination from either direct or indirect transfer of DNA. To investigate potential DNA transfer between items stored within the same evidence package, we conducted a simulation study with items commonly encountered during forensic casework. **Methods:** Participants were grouped in pairs, each of them handling the same type of item to simulate the activity conducted at the crime scene. The items were then collected from each pair of participants and stored in the same evidence package for 4 to 5 days. To evaluate the basal DNA transfer between items within the same package, the packed items were not subjected to friction, force, or long-distance movement in this study. **Results:** We have observed the occurrence of DNA transfer on 39% of the studied items inside the package, which changed the source attribution of the DNA profiles for 10% of the recovered samples. Our results showed that the types of items were associated with the number of transferred alleles and the amount of DNA recovered, while no association was found between the number of transferred alleles and the amount of DNA on the studied items. **Conclusions:** Taken together, the results from this study reiterate the importance of packing each item from the crime scene separately, especially when packing items together may impact the interpretation of source attribution.

## 1. Introduction

Biological samples with low levels of DNA have sometimes been referred to as “trace DNA” as their specific source cannot be determined, or “touch DNA” as “touching” is assumed to be the activity that left these samples behind.

DNA deposited by “touch” is often assumed to come from cells sloughed off from the outermost layer of skin [1]. Attributed to the active process of desquamation, an adult human can shed as many as 1000 cells/cm^2^/hour in a continuous manner, leading to the loss of up to 1 billion corneocytes from the body each day [2].

Despite the large number of shed cells, the recovery of full DNA profiles from fingerprints, a typical source of touch DNA, was first demonstrated only in 1997 in the seminal paper by Van Oorschot and Jones [3]. Even with this ground-breaking discovery and the subsequent progress in touch DNA studies, it was still challenging to apply earlier DNA profiling technologies to solve criminal cases involving touch DNA.

This challenge likely arose due to a combination of two aspects of touch DNA. Firstly, most of the shed cells may be anucleate, as the outermost layer of the epidermis consists of fully differentiated keratinocytes which have lost their nuclei during the keratinization process [4]. Therefore, detectable DNA may only come from a limited number of nucleated epithelia, free nuclei from degraded cells, and cell-free DNA. Secondly, the amount of DNA deposited onto an item through touching or handling can be affected by various factors, such as duration of contact, nature of contact, and the shedder status of an individual. These factors may explain the wide range of touch DNA—from 0 ng to 75 ng—that has been reported to be recovered from items after regular use by an individual [5] and the difficulty in obtaining an interpretable profile from touch DNA with quantities at the lower range. With recent improvements in DNA profiling technologies, such as the use of newer DNA analysis kits and genetic analyzer platforms, the sensitivity of DNA detection and the probability of obtaining interpretable DNA profiles from touch DNA samples have been dramatically enhanced.

However, these improvements in DNA analysis would also lead to the detection of small amounts of transferred DNA unrelated to the crime, such as background DNA deposited by activities prior to the crime or other DNA introduced through secondary DNA transfer. Nonetheless, this transferred DNA would complicate inferences that can be drawn from the DNA evidence. Hence, it is also necessary to address the probability of DNA transfer when evaluating the DNA evidence.

DNA transfer can occur when two items are placed together with direct contact. This may happen during the evidence collection process when multiple items from the crime scene are packed and transported together. Previous studies have reported the possibility of DNA transfer from a packed item to the interior of the evidence packaging, as well as between items stored within the same package [6]. However, several of these studies only examined DNA transfer using biological fluids with high levels of DNA, such as blood and saliva, while other studies involving touch DNA only evaluated the DNA transfer from the packed item to the interior of the packaging, but not amongst the items themselves [7].

In this study, we have assessed the occurrence of DNA transfer between two items that had been packed within the same package, a scenario that can occur during the handling and packing of evidence items by law enforcement. This study was conducted in response to a real operational issue, which led to a need to inform both law enforcement and the courts what can happen when exhibits are packed and stored together. As such, we focused on evaluating the basal rate of DNA transfer when items were packed together instead of mimicking the friction, temperature, and movement involved during long-distance transport of the packaged items from the crime scene to the laboratory.

## 2. Materials and Methods

### 2.1. Preparation of Mock Casework Items

Nine types and a total of 94 items commonly encountered in casework were used in this study (Table 1). These items were divided into two groups to study DNA transfer pertaining to touch DNA and/or saliva. All items, except paper and cigarette butts, were rendered ‘DNA-free’ by soaking in 1 g/L sodium hypochlorite for 2 h, followed by soaking in ultrapure water (Milli-Q water; 18.2 MΩ·cm resistivity) (Merck Millipore, Burlington, MA, USA) for 4 h.

To assess if DNA was present on the items prior to conducting this study, the surfaces of randomly selected items for each type, except cigarette butts, were swabbed and processed as described in Section 2.3. Additionally, one 15 × 11 cm paper envelope, three 24 × 16 cm paper envelopes, and one 31 × 21 cm paper envelope, which were used as packaging for the mock casework items, were also randomly selected, and the interior surfaces of these paper envelopes were swabbed and processed as described in Section 2.3.

### 2.2. Experimental Design

**Figure 1:**
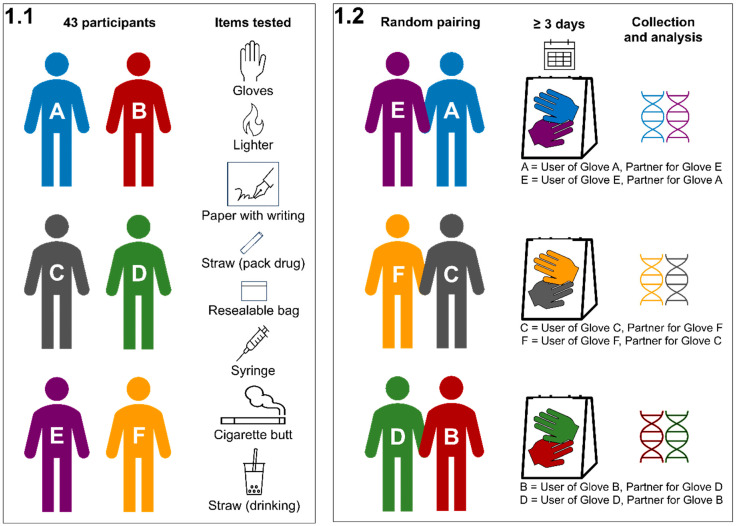
Experimental design for studying possible DNA transfer between items packed within a single evidence package. (1.1) Participants were requested to individually handle a list of commonly encountered casework items to simulate handling at a crime scene. The items included gloves, plastic straws used for packing drugs, lighters, paper with writing, syringes, cigarette butts, and plastic straws used for drinking. (1.2) After handling, each participant was randomly paired with another participant, with whom they had no contact before and after the item handling. As per the illustration above, two gloves, one from each participant, were collected and placed together into one envelope to mimic multiple items packed into a single evidence package. The items were kept together for 4 or 5 days before the DNA was collected from the items and analyzed. Each participant is simultaneously deemed a “user” and a “partner” in each of the experiments, with the “user” being defined as having direct contact with the item (e.g., glove) and as a “partner” as having only indirect contact with the item. For example, Contributor A (blue) is a “user” of Glove A since he has directly worn and used the glove. On the other hand, Contributor A is a “partner” for Glove E (purple) as he had no direct contact with Glove E, and any traces of Contributor A’s DNA on Glove E would only be possible through DNA transfer from Glove A to E during storage within the same evidence packaging. For resealable bags, straws (touch), paper, and syringes, participants were requested to refrain from washing their hands at least 1 h before the experiment to ensure that as much DNA as possible would be deposited on the items.

This study sought to examine the occurrence of DNA transfer between casework items that may have been packed together into a single package (graphically summarized in Figure 1).

Two identical items (e.g., gloves) were allocated to a pair of participants. Each of the 43 participants independently used their allocated item in a manner that could mimic its utility in a crime event (Table 1). The items were then collected from the pair of participants and placed into a single envelope package for 4 or 5 days.

In this design, each participant is concurrently a “user” (i.e., participant had direct contact/handling of one item) and a “partner” (i.e., participant’s DNA was transferred to the other item through co-placement of both items in a common package).

DNA samples were separately collected from all exposed surfaces of each item via ‘double-swabbing’ with wet and dry sterile cotton swabs, with the exception of cigarette butts. For cigarette butts, 1 cm from the unburnt end of the tipping paper was cut. All samples were collected 4 days after they were placed in the envelope package, except for all latex gloves, 10 cotton gloves, and 6 plastic straws for drinking, which were collected 5 days after being placed in the envelope package. The swabs and cuttings from cigarette butts were stored in microcentrifuge tubes at −21 °C prior to extraction.

Informed consent was obtained from each participant involved in this study, and DNA profiles were obtained from each individual as references. This study has been approved by the Domain Specific Review Board of the National Healthcare Group, Ministry of Health, Singapore.

### 2.3. DNA Processing

DNA extraction was performed using the DNA IQ™ Casework Extraction Kit and the DNA IQ™ Casework Pro Kit on a Maxwell^®^ FSC instrument (Promega Corporation, Seoul, Republic of Korea) as per the manufacturer’s protocol [8,9]. DNA yield was estimated using Quantifiler^®^ Trio DNA Quantification kit (Applied Biosystems, Warrington, UK) on the QuantStudio™ 7 Flex Real-Time PCR System (Applied Biosystems, Singapore) as per the manufacturer’s protocol [10,11]. STR-PCR amplification was performed (29 cycles) with a GlobalFiler™ PCR Amplification kit (Applied Biosystems, Warrington, UK), as per the manufacturer’s protocol [12], using 1 ng input DNA or a maximum of 15 µL DNA extract. Capillary electrophoresis was performed on the 3500 xL Genetic Analyzer (Applied Biosystems, Ibaraki, Japan) with 3 µL of amplified product injected at 1.2 kV for 24 s. Results were analyzed using GeneMapper^®^ ID-X v1.2 software.

### 2.4. Profile Analyses

DNA profiles were interpreted with an analytical threshold of 110 RFU, a stochastic threshold of 535 RFU, and the number of contributors determined based on the method used by Szkuta et al. [13]. DNA profiles of the contributors were deemed reportable only if they met the laboratory’s minimum reporting criteria of 16 alleles before the likelihood ratio (LR) was calculated using EuroForMix v3.2. The DNA mixture profiles were deemed to have major and minor contributors when EuroForMix deconvolution showed the major contributor proportion to be at least 75% of the total DNA contribution. DNA profiles with four or more contributors were deemed uninterpretable, and no LR was calculated.

Interpreted profiles were then compared with the reference profiles of the participants. Alleles detected were first attributed to the “user” participant who had directly used the item, followed by the paired “partner” participant whose item had been co-placed into the same packaging. Alleles that could not be attributed to either the “user” or the “partner” were termed as foreign alleles.

### 2.5. Statistical Analyses

A Kruskal–Wallis test was performed on the association between item type and the above-mentioned variables. *p*-value < 0.05 was considered statistically significant. Post-hoc analysis (Dunn’s Test) was also performed, followed by Bonferroni Correction.

## 3. Results

### 3.1. DNA Recovery from Different Types of Items and Biological Materials

A total of 94 samples were recovered from nine types of items (Table 1). These items were divided into two groups based on the different biological materials recovered. Touch DNA was collected from items in Group A (cotton gloves, latex gloves, lighters, paper, plastic straws for drug packing, resealable bags, and syringes) while a mix of both saliva and touch DNA was concurrently collected from items in Group B (cigarette butts and plastic straws for drinking).

**Figure 2:**
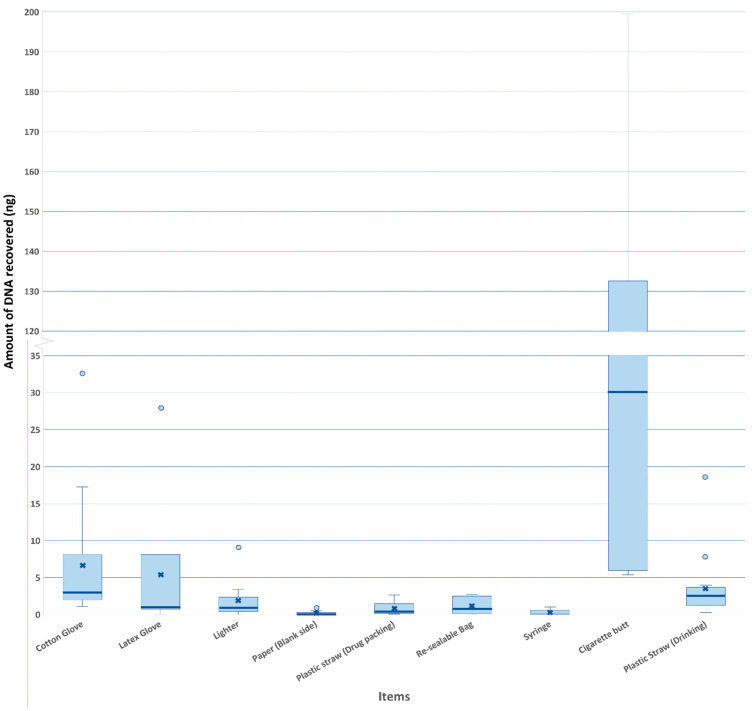
Amount of DNA recovered from different types of items. DNA quantities from nine items and the respective mean (represented by bold x), median (bold line), and outlier (blue circle).

The amount of DNA recovered from the various types of items is shown in Figure 2. Touch DNA recovered from items in Group A ranged from 0 ng to 32 ng, with a median of 3 ng. In comparison, items in Group B yielded more DNA, with the median DNA amount recovered from cigarette butts and plastic drinking straws being 30 ng and 2.5 ng, respectively.

### 3.2. Recovery of Alleles That Could Be Attributed to the “User” and the “Partner”

**Figure 3:**
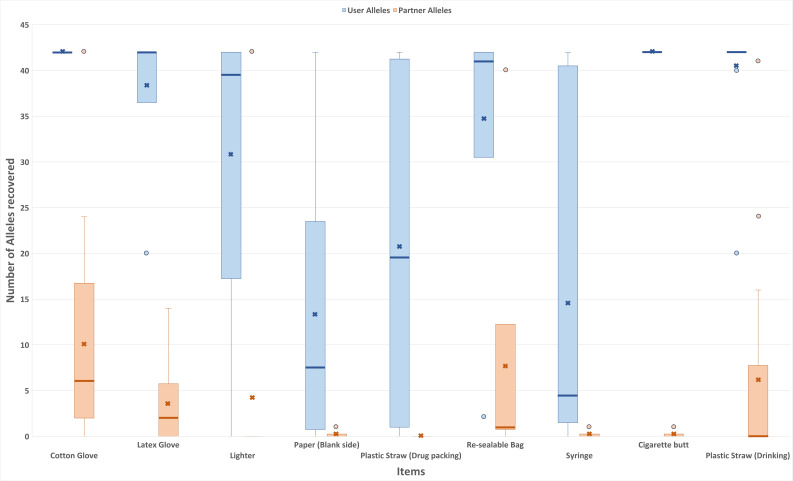
Number of DNA alleles recovered from different types of items. Number of “user” and “partner” alleles observed on nine items and the respective mean (represented by bold x), median (bold line), and outlier (blue circle and orange circle).

The occurrence of DNA transfer between the two items within the same package was observed in a total of 37 items (39% of the total number of items) (Table 2). To evaluate this transfer, the number of alleles attributed to either the “user” or the “partner” was compared (Figure 3).

It was observed that the number of alleles that can be attributed to the “user” was generally in the higher range of 20 alleles and above for all the studied items (Figure 3). In contrast, it was observed that the number of alleles that can be attributed to the “partner” was generally lower than 20 alleles, except for the outliers seen in those samples collected from cotton gloves, lighters, resealable bags, and plastic straws for drinking.

Significant differences were found in the amount of DNA, number of alleles attributed to the “user”, and number of alleles attributed to the “partner” among different types of items (Table 3).

### 3.3. Interpretation of DNA Profiles

All the DNA profiles were subsequently interpreted, and the interpretation results are shown in Table 4. Reportable DNA profiles were obtained from a total of 72 items (77%). The majority of these profiles were attributed to the “user” (62 out of 72 items—86%); it should, however, be noted that only 33 (53%) of them were single-source, while the remaining 29 (47%) exhibited additional alleles that could be attributed to the “partner”. However, based on our laboratory criterion of a minimum of 16 alleles for reporting, the recovery rate of reportable DNA profiles from the “partner” was relatively low at 10% (Table 4; 1 attributed to the "partner" plus 8 attributed to a mix of the “user” and the “partner” making up 9 out of 94 items).

The source attribution of reportable DNA profiles recovered from different types of items is detailed in Table 5. A total of eight DNA mixtures comprising “partner” and “user” alleles were recovered from four cotton gloves, one resealable bag, and three plastic straws for drinking, while one DNA profile attributed to the “partner” was recovered from a lighter. Interestingly, one DNA mixture was recovered from a plastic straw for drug packing, whereby alleles of a foreign person were found together with the “user”.

For items directly handled by “users”, a total of 71 “users” could be included as single contributors or major contributors. The allele counts ranged from 18 to 42 with likelihood ratio (LR) values between 3.15 × 10^5^ and 9.3 × 10^35^, respectively. In contrast, for items that had not been directly handled by the “partner”, 9 “partners” could be included as major or minor contributors. In these cases, the allele counts (16 to 42) and LR values (3.83 × 10^4^ to 3.01 × 10^27^) were generally lower. As expected, the majority (64 out of 71) of the “user” LRs were in the quintillions or higher, while only a minority (three out of nine) of the “partner” LRs exceeded the quintillion mark. Nevertheless, the allele counts and LR values of the “partner” still provided strong evidence to support their inclusion as a contributor (Supplementary Materials).

## 4. Discussion

### 4.1. The Amount of DNA Does Not Correlate with the Number of Transferred Alleles

In this study, the amounts of DNA recovered from cigarette butts were the highest among the items, which was possibly due to saliva (with high levels of DNA) being deposited onto the cigarette butts. Although saliva was likely present on the plastic straw for drinking, relatively low levels of DNA were obtained. This could possibly be attributed to the duration of direct contact between the cigarette butts and the mouth of the participant being longer than that of the straws, which may have led to the deposition of higher amounts of biological samples on the cigarette butts. Another factor could be the porosity of the cigarette butt, possibly absorbing/trapping the DNA, unlike the straw, where the DNA could have been easily wiped off. It should also be noted that DNA extraction of cigarette butts and plastic straws was different in that DNA was extracted directly from cigarette butt paper, vis-à-vis plastic straws, where the DNA extraction was indirect (i.e., from the wet-dry swabs of the straw). As such, the DNA extraction efficiency from the cigarette butts may also be higher, thereby giving rise to a higher DNA yield. Although a much higher amount of DNA was recovered from the cigarette butt than from the rest of the items, the number of transferred alleles (alleles attributed to the “partner”) between the cigarette butts in the same package was observed to be similar to that from the lighters and plastic straw for drug packing (Figure 3), from which only a relatively low amount of DNA was recovered (Figure 2). As such, no obvious correlation between the amount of DNA and the number of transferred alleles could be determined, which suggested that DNA transfer between items may occur in similar ways regardless of the amount of DNA present.

### 4.2. DNA Transfer Between Different Types of Items

Our results showed that the majority of the reportable DNA profiles could be attributed to the “user”, which is expected as direct contact with or handling of an item may deposit more DNA onto it than that from the secondary DNA transfer in the setting of our study.

For five types of items—cotton gloves, latex gloves, lighters, resealable bags, and plastic straws for drinking—the number of alleles that could be attributed to the “partner” (between 0 and 24 alleles, except for the outliers) was comparable with the range of transferred alleles detected in a previous study [6]. With respect to cigarette butts, Goray et al. reported observing 1–5 transferred alleles in 37.9% of the samples and 6–10 transferred alleles in 62.1% of the samples [6]. In comparison, our study showed fewer transferred alleles between the cigarette butts, possibly because our study only assessed the basal DNA transfer with shorter distance movement of the evidence package, while the settings in their study involved longer distance movement, friction, and force. A similar low transfer rate was observed by Thornbury et al. [14] when they studied DNA transfer without contact from dried biological materials on different objects. For all objects subjected to tapping action, the highest transfer rate of saliva was one out of six, while no transfer of touch DNA was observed.

The number of transferred alleles between plastic straws for drinking was observed to be higher than that of the cigarette butts, which indicated that DNA transfer may be more likely to occur between non-porous surfaces than porous surfaces, in corroboration with a previous report [15].

In the study by Stella et al. [7], where non-porous polyvinyl chloride (PVC) capped tubes were used to mimic real exhibits, 100% (three out of three tubes) of saliva and 0% (zero out of three tubes) of touch DNA were transferred from one part of the tube to another part of the tube through the interior of the package as the intermediate surface. Their results contrasted with our study involving items often encountered in crime casework—19% (3 out of 16 plastic straws for drinking) of saliva were transferred to the paired items, and 25% (4 out of 16 cotton gloves), 10% (1 out of 10 lighters), and 17% (1 out of 6 resealable bags) of touch DNA was transferred to the paired items. The difference could likely be attributed to the amount of saliva/touch DNA used in their study. In the case of saliva, Stella et al had used 100 µl (roughly two droplets) of saliva to spot the tube, which would be substantially more than what would remain on a straw used for drinking. With respect to the touch DNA, Stella et al. had participants rub an area on the tube with a fingertip, which would likely have deposited less biological material compared to our study, where participants handled a larger surface area, e.g., wearing a glove for hours, holding a lighter while rolling the wheel, and using a syringe.

In general, there were significant differences in the amount of DNA recovered, as well as the number of alleles attributed to the “user” and “partner” amongst the different types of items used in this study. This suggests that item type may be associated with the number of transferred alleles between two items.

### 4.3. DNA Transfer Within the Same Package Could Be a Potential Source of Contamination

In this study, DNA transfer between the two items within the same evidence package was observed in 39% of the recovered samples (Table 2; 37/94 samples). Although the majority of the interpretable DNA profiles obtained could be attributed to the “user”, 46% of these profiles also contained additional alleles that could be attributed to the “partner” (although it was below our laboratory reporting threshold). Moreover, the number of transferred alleles in this study may be underestimated as shared alleles can exist between the “user” and the “partner”. In this respect, adoption of massively parallel sequencing (MPS) techniques (compared to traditional STR genotyping via capillary electrophoresis techniques) may enable better distinction between the “user” and “partner” in a mixed DNA sample even if they share the same number of STR repeats [16,17]. Nevertheless, these results indicate that secondary DNA transfer within the same evidence package can be a real source of contamination in criminal casework. In addition, other factors commonly encountered during the evidence collection process may further increase the probability of DNA transfer within the package, such as long-distance movement and friction amongst the items packed together [18].

### 4.4. DNA Transfer May Further Interfere with Downstream DNA Profile Interpretation

Transfer of DNA between two items can alter the DNA profile and consequently impact the interpretation of source attribution. The impact of DNA transfer occurring within the evidence package on the interpretation of source attribution in our study was evaluated. While the interpretation of source attribution was unaffected for most of the 72 items with reportable DNA profiles, likely a reflection of the generally low levels of DNA being transferred, there were changes in the interpretation of source attribution for nine items, a non-trivial 12.5% (Table 6). This represents 10% of the items (Table 6; 9/94 samples) and indicates that DNA transfer can indeed influence the interpretation of source attribution, especially when the initial DNA on the item is in minute quantities. Additionally, any further DNA lost from these minute quantities due to transfer may impede our attempt to obtain an interpretable DNA profile from the item.

This issue of influence on the interpretation of source attribution may be addressed by evaluative reporting, which is a recent trend in DNA transfer studies [19,20,21]. Our present study shares similarities in the investigation of the frequency of detecting the person of interest, such as the “user” and “partner”. Such frequencies may then be used towards evaluating the likelihood of direct and indirect transfer when items have been packed together, though it should be noted that scenarios involving different items packed together may require further studies.

Furthermore, the current study was focused on the basal rate of DNA transfer between items packed together, as it was in response to an operational issue and the need to inform both law enforcement and the courts what can happen when exhibits of the same type are packed and stored together. As such, it does not address variables such as friction between items, environmental temperature, and movement of items within the package during long-distance transport of the packaged items from the crime scene to the laboratory. It also does not address the issue of the directionality of DNA transfer between different types of items packed in the same package.

## 5. Conclusions

The present study highlighted the general DNA recovery and the number of “user” and “partner” alleles from the different types of items. With more questions being raised in courts about DNA transfer, e.g., activities that can lead to DNA deposition, quantities of DNA deposited, and associated probabilities of the deposited DNA being detectable, it is timely and relevant to assess the impact of DNA transfer occurring when multiple evidential items are packed in a common package. This study showed that DNA transfer was observed on 39% of the items (refer to Section 4.3 for details) that had been packed together, with 10% of the items (refer to Section 4.4 for details) having altered interpretations (e.g., ‘false inclusion’ on the source attribution). As such, laboratories and law enforcement agencies would benefit from procedures guiding the individual packaging of each evidence item to reduce the risk of secondary DNA transfer within an evidence package, leading to an inaccurate source attribution. One approach may be to pack each evidence item separately unless the items were found to already be in contact with each other upon discovery. Another approach could be to collect DNA from the evidence items at the crime scene itself to minimize potential DNA transfer while transporting the evidence items to the laboratory.

## Figures and Tables

**Table 1:** List of different types of items used in this study. Number of each item in groups A and B, and the way they were used.

Item	N	Way of Use	Note
Group A			
Cotton glove	16	Worn for whole day for ≥2 days	Interior of glove facing out when placed in envelope
Latex glove	6	Worn for ≥1 h	Interior of glove facing out when placed in envelope
Lighter	10	Held for 1 min and rolled the wheel at least 3 times every hour for 6 h	
Paper	10	Wrote 3 sentences on it (landscape) and folded it twice, with each fold bringing shorter ends to each other, unfolded, and pressed down on the table (blank side in contact with the table) to mimic a debt collector pressing the paper onto the door of the debtor’s house before fixing it with adhesive tape.	Folded form when placed in envelope
Plastic straw for drug packing	10	Cut and heat sealed	
Re-sealable bag	6	Touched ≥ 5 times within a day	
Syringe	10	Opened cap and plunged 3 times, then capped back	
			
Group B			
Cigarette butt	10	Held as when smoking	Provided by participants after smoking
Plastic straw for drinking	16	Used to drink beverage	
Total	94		

**Table 2:** Number of items with at least one allele that could be attributed to the “partner”. Count and percentage of each item type from Group A (items with touch DNA) and Group B (items with both touch DNA and saliva) with one or more alleles that could be attributed to the “partner”.

Group	Item Type	Count (% of Total Number of Each Item Type)
A	Cotton glove (n = 16)	14 (88%)
	Latex glove (n = 6)	4 (67%)
	Lighter (n = 10)	1 (10%)
	Paper (n = 10)	2 (20%)
	Plastic straw for drug packing (n = 10)	0 (0%)
	Resealable bag (n = 6)	5 (83%)
	Syringe (n = 10)	2 (20%)
B	Cigarette butt (n = 10)	2 (20%)
	Plastic straw for drinking (n = 16)	7 (44%)

**Table 3:** Items with significant differences in the amount of DNA, number of alleles attributed to “user” or number of alleles attributed to “partner” after Kruskal–Wallis test and post-hoc analysis (Dunn’s Test) with Bonferroni Correction.

Category of Comparison	Item (A)	Item with Significant Difference with Item (A)
Amount of DNA	Cotton glove	Paper
		Plastic straw for drug packing
		Syringe
	Cigarette butt	Lighter
		Paper
		Plastic straw for drug packing
		Resealable bag
		Syringe
	Plastic straw for drinking	Paper
		Syringe
Number of alleles attributed to “user”	Cotton glove	Paper
		Plastic straw for drug packing
		Syringe
	Cigarette butt	Paper
		Plastic straw for drug packing
		Syringe
	Plastic straw for drinking	Paper
		Plastic straw for drug packing
		Syringe
Number of alleles attributed to “partner”	Cotton glove	Lighter
		Paper
		Plastic straw for drug packing
		Syringe
		Cigarette butt

**Table 4:** Interpretation results of DNA profiles. Count and percentage of the reportable, not reportable, and uninterpretable profiles. Reportable profiles were further classified into sub-categories depending on attribution.

Interpretation	Count of Sample
Reportable	72 (76.6%)
• Attributed to the “user”	62
• Attributed to the “partner”	1
• Mix of the “user” and the “partner”	8
• Mix of the “user” and foreign person	1
Not reportable	21 (22.3%)
Uninterpretable	1 (1.1%)

**Table 5:** Attribution of reportable DNA profiles from items grouped by different types. Count and percentage of profile attribution for each item type.

Item Type	Attributed to the “User”	Attributed to the “User” and the “Partner”	Attributed to the “Partner”	Attributed to the “User” and Foreign Person
Cotton glove (n = 16)	11 (69%)	4 (25%)		
Latex glove (n = 6)	6 (100%)			
Lighter (n = 10)	8 (80%)		1 (10%)	
Paper (n = 10)	3 (30%)			
Plastic straw for drug packing (n = 10)	4 (40%)			1 (10%)
Resealable bag (n = 6)	4 (67%)	1 (17%)		
Syringe (n = 10)	3 (30%)			
Cigarette butt (n = 10)	10 (100%)			
Plastic straw for drinking (n = 16)	13 (81%)	3 (19%)		

**Table 6:** Changes in DNA profile interpretation of source attribution caused by DNA transfer. Count of the number of items for each of the three different ways the interpretation of source attribution has been altered by DNA transfer.

Number of Items	Expected Profile If No DNA Transfer	Observed Profile
1	Not reportable single-source profile of the “user” but less than 16 alleles	“partner” being the major contributor
3	Essentially a single-source profile of the “user”	“user” and “partner” being approximately equal co-contributors
5	Essentially a single-source profile of the “user”	“user” being the major contributor and “partner” being the minor contributor

## Data Availability

Raw electropherograms are not available due to restrictions on privacy, legal, or ethical reasons.

## Supplementary Materials

The following supporting information can be downloaded at: https://www.mdpi.com/article/10.3390/genes16080894/s1. Refer to Excel spreadsheet S1: Table of Raw Data.

## References

[1] Wiegand P., Kleiber M. (1997). DNA typing of epithelial cells after strangulation. Int. J. Legal Med..

[2] Roberts D., Marks R. (1980). The determination of regional and age variations in the rate of desquamation: A comparison of four techniques. J. Investig. Dermatol..

[3] van Oorschot R.A., Jones M.K. (1997). DNA fingerprints from fingerprints. Nature.

[4] Bragulla H.H., Homberger D.G. (2009). Structure and functions of keratin proteins in simple, stratified, keratinized and cornified epithelia. J. Anat..

[5] Meakin G., Jamieson A. (2013). DNA transfer: Review and implications for casework. Forensic Sci. Int. Genet..

[6] Goray M., van Oorschot R.A., Mitchell R.J. (2012). DNA transfer within forensic exhibit packaging: Potential for DNA loss and relocation. Forensic Sci. Int. Genet..

[7] Stella C.J., Meakin G.E., van Oorschot R.A.H. (2022). DNA transfer in packaging: Attention required. Forensic Sci. Int. Genet. Suppl. Ser..

[8] Promega Corporation. DNA IQTM Casework Pro Kit for Maxwell® 16 Technical Manual Part# TM332, Revised 11/11.

[9] Promega Corporation. Maxwell® FSC Instrument Operating Manual TM462 Revised 8/17.

[10] Applied Biosystems. QuantStudio™ 6/7 Flex Real Time PCR System, Getting Started Guide, Rev A, 2013.

[11] Applied Biosystems. Quantifiler™ HP and Trio DNA Quantification Kits, User Guide, 2017.

[12] Applied Biosystems. GlobalFiler™ PCR Amplification Kit, User Guide, July 2016.

[13] Szkuta B., Ballantyne K.N., van Oorschot R.A.H. (2017). Transfer and persistence of DNA on the hands and the influence of activities performed. Forensic Sci. Int. Genet..

[14] Thornbury D., Goray M., van Oorschot R.A.H. (2021). Indirect DNA transfer without contact from dried biological materials on various surfaces. Forensic Sci. Int. Genet..

[15] Goray M., Mitchell R.J., van Oorschot R.A. (2010). Investigation of secondary DNA transfer of skin cells under controlled test conditions. Leg. Med..

[16] Gettings K.B., Kiesler K.M., Faith S.A., Montano E., Baker C.H., Young B.A., Guerrieri R.A., Vallone P.M. (2016). Sequence variation of 22 autosomal STR loci detected by next generation sequencing. Forensic Sci. Int. Genet..

[17] Neste C.V., Nieuwerburgh F.V., Hoofstat D.V., Deforce D. (2012). Forensic STR analysis using massive parallel sequencing. Forensic Sci. Int. Genet..

[18] Goray M., Eken E., Mitchell R.J., van Oorschot R.A. (2010). Secondary DNA transfer of biological substances under varying test conditions. Forensic Sci. Int. Genet..

[19] van Oorschot R.A.H., Meakin G.E., Kokshoorn B., Goray M., Szkuta B. (2024). DNA Transfer in Forensic Science: Recent Progress towards Meeting Challenges. Genes.

[20] Cahill A., Volgin L., van Oorschot R.A.H., Taylor D., Goray M. (2024). Where did it go? A study of DNA transfer in a social setting. Forensic Sci. Int. Genet..

[21] Taylor D., Volgin L., Kokshoorn B. (2024). Accounting for site-to-site DNA transfer on a packaged exhibit in an evaluation given activity level propositions. Forensic Sci. Int. Genet..

